# Challenges for Super-Resolution Localization Microscopy and Biomolecular Fluorescent Nano-Probing in Cancer Research

**DOI:** 10.3390/ijms18102066

**Published:** 2017-09-28

**Authors:** Michael Hausmann, Nataša Ilić, Götz Pilarczyk, Jin-Ho Lee, Abiramy Logeswaran, Aurora Paola Borroni, Matthias Krufczik, Franziska Theda, Nadine Waltrich, Felix Bestvater, Georg Hildenbrand, Christoph Cremer, Michael Blank

**Affiliations:** 1 Kirchhoff-Institute for Physics, University of Heidelberg, Im Neuenheimer Feld 227, 69120 Heidelberg, Germany; goetz.pilarczyk@kip.uni-heidelberg.de (G.P.); jin-ho.lee@kip.uni-heidelberg.de (J.-H.L.); abiramy_l@hotmail.com (A.L.); krufczik@kip.uni-heidelberg.de (M.K.); f.theda@gmx.de (F.T.); nadine.waltrich@googlemail.com (N.W.); hilden@kip.uni-heidelberg.de (G.H.); 2 Laboratory of Molecular and Cellular Cancer Biology, Faculty of Medicine, Bar-Ilan University, 8 Henrietta Szold ST, Safed 1311502, Israel; natasa.ilic@biu.ac.il (N.I.); aurorapaola.borroni@gmail.com (A.P.B.); 3 German Cancer Research Center (DKFZ), Im Neuenheimer Feld 280, 69120 Heidelberg, Germany; f.bestvater@dkfz.de; 4 Department of Radiation Oncology, Universitätsmedizin Mannheim, University of Heidelberg, Theodor-Kutzer-Ufer 3-5, 68159 Mannheim, Germany; 5 Institute of Molecular Biology, Ackermannweg 4, 55128 Mainz, Germany; C.Cremer@imb-mainz.de

**Keywords:** fluorescent nano-probes, super-resolution localization microscopy, receptor conformation changes, chromatin organization, chromatin radiation response, cancer research, Smurf2, γ-H2AX phosphorylation sites

## Abstract

Understanding molecular interactions and regulatory mechanisms in tumor initiation, progression, and treatment response are key requirements towards advanced cancer diagnosis and novel treatment procedures in personalized medicine. Beyond decoding the gene expression, malfunctioning and cancer-related epigenetic pathways, investigations of the spatial receptor arrangements in membranes and genome organization in cell nuclei, on the nano-scale, contribute to elucidating complex molecular mechanisms in cells and tissues. By these means, the correlation between cell function and spatial organization of molecules or molecular complexes can be studied, with respect to carcinogenesis, tumor sensitivity or tumor resistance to anticancer therapies, like radiation or antibody treatment. Here, we present several new applications for bio-molecular nano-probes and super-resolution, laser fluorescence localization microscopy and their potential in life sciences, especially in biomedical and cancer research. By means of a tool-box of fluorescent antibodies, green fluorescent protein (GFP) tagging, or specific oligonucleotides, we present tumor relevant re-arrangements of Erb-receptors in membranes, spatial organization of Smad specific ubiquitin protein ligase 2 (Smurf2) in the cytosol, tumor cell characteristic heterochromatin organization, and molecular re-arrangements induced by radiation or antibody treatment. The main purpose of this article is to demonstrate how nano-scaled distance measurements between bio-molecules, tagged by appropriate nano-probes, can be applied to elucidate structures and conformations of molecular complexes which are characteristic of tumorigenesis and treatment responses. These applications open new avenues towards a better interpretation of the spatial organization and treatment responses of functionally relevant molecules, at the single cell level, in normal and cancer cells, offering new potentials for individualized medicine.

## 1. Introduction

### 1.1. Challenges of Cancer Research

Cancer is one of the leading causes of morbidity and mortality in the developed world, killing more than eight million people annually, worldwide. Despite significant advances being made in the treatment of specific cancer types, eradication of the disease, especially in its most dangerous metastatic forms, has yet to be achieved. Recent progress in cancer research has established a consequential link between compromised genomic integrity, altered gene expression, impaired DNA damage response, and dysregulated membrane receptor activation and trafficking. These components form the main driving forces in carcinogenesis, and evidently, determine the therapeutic outcomes in many types of cancer [1,2,3,4,5,6,7,8,9,10,11,12,13]. However, little is known about the mechanisms spanning through and interlocking these components. This dearth of knowledge creates significant gaps in our understanding of the mechanisms operating in, and leading to, cancer initiation and progression, and the sensitivity of tumor cells to anticancer therapies, and impedes the design of novel, more efficient, therapeutic approaches to cure cancer. 

To address these challenges, we need better tools, especially those which allow better insights, not only into the micro-compartmentalization, but also into the nano-structural organization of molecules in the cell, and thus into the spatio-temporal organization and dynamics of cancer-associated pathways, occurring within a three dimensional (3D) cellular space. 

During the last decades, novel sophisticated laser techniques have become indispensable for applications in life sciences. For visualization of cells, cell nuclei, or other subcellular components, 3D confocal laser scanning microscopy has become the standard technique. With the advantage of specific labeling and multi-color detection of fluorescence, a new understanding of cells and single cell analysis has been developed. Nevertheless, the detection of arrangements, movements, and interactions of single molecules and the understanding of the mechanisms behind these processes are still challenging and require both precise instrumentation and highly-specific nano-probes. Pointillist visualization, by means of localization microscopy [14,15], provides a resolution level on which functionally discontinuous processes between molecules are not optically merged into an apparently continuous structure–function correlation. Moreover, this technique can be applied in such a way that images and image processing can be circumvented and structuring principles can be obtained from point-to-point distance frequency distributions. This knowledge may provide a better understanding of how molecules spatially interact and how changes in this spatial interaction may contribute to cell transformation into a malfunctional entity, resulting in tumor generation and cancer progression. 

### 1.2. Localization Microscopy

Novel approaches in laser light microscopy circumventing the Abbe–Rayleigh boundary conditions of diffraction, enable effective optical resolutions down to, for instance, single nucleosomes, antibodies, or receptors—which means a scale in the order of 10 nm. Beyond sophisticated techniques of point-spread function engineering [16,17], methodological approaches using standard objective lenses and instrumental equipment are often summed up as localization microscopy. These approaches are based on the fundamental concept of optical isolation of molecular labels by different spectral signatures, which means for instance constant differences in the absorption/emission spectrum [18], or by switching between two different spectral states like “on/off” (blinking) to achieve a temporal isolation and/or a spatial separation of single signals (see for instance photoactivated localization microscopy (PALM) [19], fluorescence PALM (FPALM) [20], stochastic optical reconstruction microscopy (STORM) [21], direct STORM (dSTORM) [22], spectral precision distance microscopy with physically modifiable fluorophores (SPDM_phymod_) [23], ground state depletion microscopy followed by individual molecule return (GSDIM) [24], etc.). These approaches allow the determination of signal locations, in terms of spatial coordinates and their spatial distances, even if these distances are below the Abbe–Rayleigh resolution limit, which is, in practice, about 200 nm laterally, and approximately 600 nm axially, using high numerical aperture objectives, such as those used in confocal laser scanning microscopy. 

Acquiring a time stack of hundreds of frames of the same object section, by localization microscopy, allows the detection of each molecular fluorescent “blinking event”. In such a way, acquired positions of individual fluorescent molecules can then be recorded into a matrix of coordinates of molecule loci. This matrix can be used to calculate point distance distributions from which principles of molecular arrangements can be extracted and interpreted into functions and interactions of the respective molecules. Furthermore, the loci matrix allows the construction of an artificial, pointillist, super-resolution image, in which the effective resolution (smallest resolvable distance between two points) depends on the lateral and axial localization precision. The latter are mainly determined by the signal-to-background noise of the specimen analyzed. Moreover, since the image is reconstructed from the loci matrix artificially, it can encode further information, obtained from data evaluation, for instance, number of next neighbors, member of clusters, etc.

A certain embodiment of localization microscopy, used in the experiments described in this article, is based on SPDM_phymod_ [23,25]. In this technique, conventional fluorophores are switched to a “dark” state by a laser light-induced, so called “reversible photo-bleaching effect”. This blinking effect, being different from real physical photo-bleaching, can be based on the molecular structure of the dye molecule, isomeric conformation changes of proteins, micro-environmental chemical conditions (e.g., pH), dye ionization, etc. These molecular conformation changes reversibly suppress the ability of a molecule to fluoresce. From this dark state, the fluorescent dye molecules statistically return into the emission state. 

Subsequently, each of the emitting fluorophores is represented by an Airy disc-like fluorescence signal on the microscopic detector, during the time series of several hundred frames. The center of mass (barycenter) of such an Airy disc approximates to a high accuracy, the spatial position (coordinates) of the emitting molecule. Typically, a localization precision, and a point-to-point distance resolution in the order of 10–20 nm, can be obtained so that pointillist images (locally differing in point densities) provide structural resolution, based on the detected molecule positions. So far, receptor cluster formation [26,27], chromatin re-arrangements [28,29,30], protein trafficking [31], chromatin loop formation [32], and other important cellular processes, have been studied by this technique at a nanoscale resolution. 

### 1.3. Nano-Probing for Localization Microscopy

For localization microscopy, the molecules of interest have to be labelled with appropriate nano-tags and nano-probing techniques. These tags have to fulfil several conditions: (a) they have to be specific without minor binding sites; (b) they have to be small in order not to strongly influence the molecular arrangements in the cell; (c) the fluorescence of the tags has to be high in order to obtain a strong signal–background relation.

Beyond specific antibodies, which are nowadays commercially available in a nearly infinite multitude, dye-labeled antibodies provide a combinatorial strategy for specific staining (e.g., [26,27,30,31]). In addition, the use of fluorescent proteins (e.g., green fluorescent proteins (GFP), yellow fluorescent proteins (YFP), etc.) tagged to a particular protein of interest has been successfully applied for nano-probing of specific molecules (e.g., [28,29,30,33]).

Although these techniques have been successful, labeling of specific DNA-sequences in cell nuclei has required additional techniques that fulfill the requirements of being sequence-complementary and specific, like probes used for Fluorescence in situ Hybridization (FISH). Another requirement for probes is to be as small as possible, in order not to modify the native molecular structure being measured with nanometer precision. This means that a sequence-specific label, consisting of only a few molecules, has to be found, in order not to occupy geometrically too much space in the cell nuclei.

COMBO-FISH (COMBinatorial oligo-nucleotide FISH [34]; for review of applications see [35]) can be seen as a significant step towards fulfilling the requirements of super-resolution localization microscopy. In contrast to FISH, using Watson–Crick binding bacterial artificial chromosome (BAC), yeast artificial chromosome (YAC) or cosmid probes for instance, COMBO-FISH combines FISH with the design of highly-specific oligo-nucleotide probe sets for any given genome region in the human genome [36,37]. For labeling of a (tumor) gene target (see for instance [38]), a set of oligo-nucleotide probes of typically several 15–30 molecule oligomers can be selected only by computer search from the sequence database, without any molecular biology lab-work. The combinations of appropriate oligo-nucleotide target sites, which can consist either homopurine or homopyrimidine sequence stretches, are then selected in such a way that after excluding repetitive sequences and/or multiple clustering sequences, the resulting set exclusively co-localizes at the given target site [39,40,41]. Such a labeling set can also consist of only one probe, binding uniquely, but in high multiplicity, to a given target site [37,42]. The probes can then be synthesized either as DNA-probes [37,38,39] or with modified backbones like PNA-probes (peptide nucleic acids probes) [41,42], in such a way that they complementary bind to a single stranded target via Watson–Crick bonding, or as a triple-strand to a double stranded target, via Hoogsteen bonding (homo-pyrimidine oligo-nucleotide probes), or reverse Hoogsteen bonding (homo-purine oligo-nucleotide probes). Using low temperature conditions [43], COMBO-FISH can be combined with immunostaining procedures using specific antibodies [37].

## 2. Results and Discussion

### 2.1. Data Interpretation: From Points to Structural Organization

Any application of localization microscopy in cancer research, medical diagnoses and personalized medicine may be challenging in such a way that a point pattern obtained by loci information of individual molecules has to be translated into functionally relevant structures and organization principles. Localization microscopy is not just microscopic imaging with better resolution than so far established techniques. Localization microscopy uses pixel information from image frames to determine a matrix of molecule loci. This matrix is used as data set for further evaluation, so that the production of an image is a secondary process and primary data evaluation does not require procedures of image processing. The measurement value cloud of an experiment is most simple, and is considered as a tensor object, bundling a population of four-dimensional vectors (*a*_1_, *a*_2_, *a*_3_, *a*_4_), with each vector representing an individual data point in the localization events of the experiment.

The vector components are as follows: *a*_1_ = fluorescence intensity in continuous values or in binary notation; *a*_2_ = the *x*- and *a*_3_ = *y*-coordinates, as collected and recorded by the camera chip; *a*_4_ = an incremental tag indicating the position in time or in the corresponding slice of the time-stacked detection frames. All vector properties are defined by the onset and disappearance of a fluorescence emission burst, the utmost important event in localization microscopy. The burst is defined by the dye’s fluorescence behavior under high intense illumination but is incrementally recorded by the camera frame recording. This means that the actual temporal burst extension is not accessible, but the superposition of the fluorescence signal length, and camera recording frame rate resolution are.

In this formulation, the reduction of all signals into an *i*-value (*a*_1_), *x*-position (*a*_2_), *y*-position (*a*_3_), *t*-slice or *z*-position (*a*_4_) can be considered as vector normalization with concomitant tensor dimensionality reduction, from four to three dimensions (*x*-, *y*- and *i*-values). In principle, other components could also be used for a conclusive tensor, as shown, for instance, by the separation of photons by lifetime tuning (SPLIT) method in stimulated emission depletion (STED) microscopy [44]. This resulting tensor is subject to further data processing actions, for example, the discrimination of whether a point is part of a specimen structure or of the background. The specimen-to-background discrimination can be done by the use of intensity properties (“bright enough for being part of the labeled specimen in comparison to unlabeled controls”) or on the basis of local geometrical constraints (“near to differently labeled neighbors”, “inside a pattern of other labeled molecules”, etc.). Another example is distances that can be calculated from coordinates as Euclidian distances between the coordinates, which are scaled with object size units obtained from the pixel size of the camera and the magnification used. In order to get a representative data set, the data from the series of nuclei analyzed were merged and if necessary normalized or averaged.

Interpretation tools that are used as the basis for such decisions are most prominently algorithms, derived from Voronoi- or Ripley’s-structuring criteria [45,46]. While Voronoi-derived procedures use triangulation methods to estimate, for example, the density of bursts inside a specimen area [30], Ripley’s-based approaches estimate the burst-to-burst distances by direct measurement of distances from a central point to the bursts which are peripheral with respect to the center but not to the neighbors. The immediate access to distance values by Ripley’s approach is an argument for the preferred use of this method in contrast to the Voronoi analysis, where distances must be derived from triangular patterns in a secondary computing process compatible to image processing. On the other hand, the Voronoi approach yields immediate area based information, which must be derived from Ripley’s analysis data in a further computing procedure.

The graphical display of the Ripley’s point-to-point distance information (absolute or relative frequency histograms) without considering absolute position information, makes the discrimination between specimen structure signals and background signals accessible. The original curve shows noisy-like fluctuations due to superposition of the specimen signals, blurring of the microscope transfer function and the camera chip acquisition characteristic. In total, these parameters restrict the possible localization precision of a point-like fluorescence burst signal. The so-called localization accuracy is an acquisition parameter, limiting the spatial resolution of the acquisition process by the labeling molecules and strategy.

**Figure 1:**
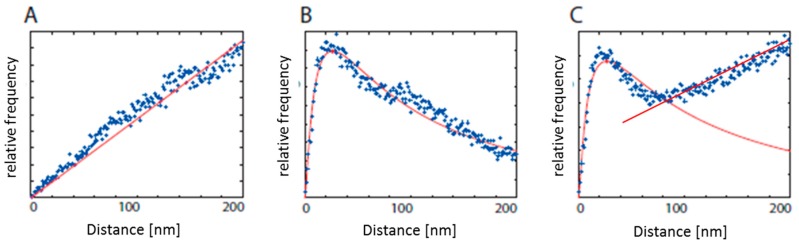
Schematic description of results obtained from localization microscopy. Blue points represent the measured frequencies of distances usually obtained from several cells of a specimen; the red curve represents the calculated envelope. The data points presented in this figure were taken from different specimens and different labels, showing the structural characteristics being described. (**A**) Distribution indicating a random arrangement of molecules; (**B**) structured organization with a characteristic dimension indicated by the maximum of the theoretical curve (red point); (**C**) organized structure implemented in randomly-distributed molecules.

The envelope function of the mentioned histogram can be divided into one or several functions which represent particular distance accumulations. In this way, elements of an organized specimen structure can be identified and subjected to further computation and classification. In addition, the appearance of spatially-disorganized background signals tends to approximate a monotonically increasing linear function (Figure 1). By removing the corresponding data points from the data cloud, an effective background reduction, without loss of specimen intensity, can be obtained. 

The specimen structure, related to non-random, local clustering, appears as individual peaks of the histogram envelope function. The maximum value of such a peak indicates the characteristic distance of a specimen structure. The symmetry and width of the peak corresponds to the molecular conformation and spatial organization of the labeled elements.

The above-mentioned considerations elucidate a particular property of localization microscopy: the microscope image in the form of a two dimensional display of a specimen structure with a certain resolution is nothing else than the visualization of the acquired vector value set. The primary data characteristic of a value cloud is that of a set of inter-value distances, with the distance histogram being the most appropriate method of visualization. The particular property of a localization microscopy result (as a cloud of isolated localized signals and their distances in between) also sheds light on the special image appearance as a non-continuous specimen structure, which appears as a pointillist-like pattern. This property is in opposition to the regular microscopy images where the specimen appears as a continuous structure. Nevertheless, one must consider that such continuity is the result of optical point spreading by resolution limitation due to diffraction. The resolution limit of conventional microscopy introduces continuity into discrete specimen structures. In this way, the uncommon specimen appearance more closely reflects the true nature of a fluorescent specimen, which consists of individual isolated and independent fluorescence localities.

This point-to-point measurement and its interpretation in structural parameters require specimen conditions that do not show any significant fluctuations in the positions of the labelling tags. Therefore, it is recommended that the specimen is fixed at a certain time point, so that a cell stage can be “frozen” for all cells on the carrier. This also makes the results comparable if the measurements are extended by hours for one specimen.

### 2.2. Cluster Formation of Epidermal Growth Factor Receptors (EGFR) and Their Response to Cancer Treatment

Receptor molecules of the epidermal growth factor (EGF) family, like ErbB2 or ErbB3, are often involved in regulatory mechanisms and thus unfortunately also in tumor induction and progression. Their expression status can be used as a prognostic factor in medical diagnosis. Modern drugs for tumor treatment, for instance, trastuzumab or pertuzumab, target such receptors and inhibit their functional pathways in order to stop uncontrolled cell growth and the formation of metastases. However, while the inhibition of ErbB2/3 regulatory pathways could affect tumor progression, its application on non-tumor normal cells may result in toxicity, including cardiac failure. Therefore, an understanding of the mechanisms behind the molecular effects of this treatment and its effects on cellular survival pathways would help to improve anti-EGFR therapies.

Simulation models [47,48,49,50] for the biophysics of receptor proteins embedded in lipid bi-layers have resulted in thermodynamically-driven receptor cluster formation, due to liquid forces and hydrophobic mismatch [50]. This process has been detected by super-resolution light microscopy and near-field optical microscopy for several receptor types. Cluster size and molecular compartmentalization became accessible by measurements in the sub-diffraction range. It was shown that cells with different expression levels of EGF-receptors (e.g., ErbB2 in breast cancer or EGFRvIII in glioblastoma) show a different frequency of clustering, and differ in cluster size and molecular arrangements of the clustering receptor molecules [26,27,46,51]. Since the cluster formation is directly correlated to functional consequences of inter-receptor communication and signaling pathways [52], it is of high interest to analyze such clusters with respect to molecular treatment.

To this end, we used the well-established breast cancer Michigan Cancer Foundation cell line 7 (MCF-7) cell model. After fixation, the cells were labelled with primary antibodies to ErbB2 and ErbB3. These antibodies were fluorescently labelled via appropriately-stained secondary antibodies. Although the cells did not show an overexpression of ErbB2 and ErbB3, an increase of ErbB2-ErbB3 heterodimers was reported [53,54].

Using localization microscopy, a cluster formation of ErbB2 and ErbB3 receptors in a less organized environment, compatible to the situation of Figure 1C, was found under different treatment conditions: (a) untreated control cells; (b) cells where the formation of ErbB2-ErbB3 heterodimers was stimulated by 15 minutes of neuregulin-1 treatment; (c) neuregulin-1 stimulated cells co-treated with trastuzumab (Herceptin^®^, Roche Diagnostics GmbH, Mannheim, Germany) for 15 min; (d) neuregulin-1 stimulated cells co-treated with trastuzumab (Herceptin^®^) for 15 min and an additional treatment with pertuzumab (Perjeta^®^, Roche Diagnostics GmbH, Mannheim, Germany) for 60 min. For each type of treatment, about 25 cells were analyzed.

**Figure 2:**
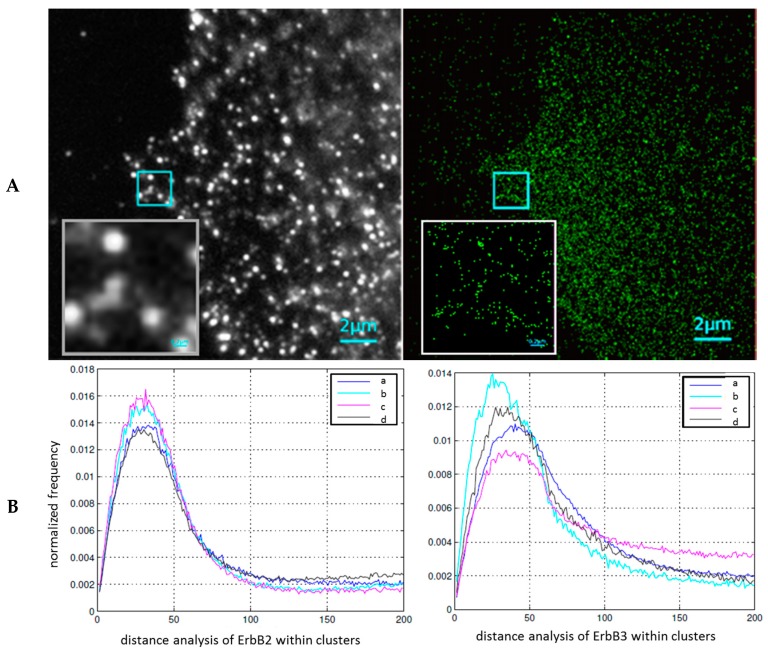
(**A**) Image section of a breast cancer cell (MCF7 cell line) after specific labelling of ErbB3: Widefield image (**left**), image obtained from localization microscopy data (**right**). The inserts are magnifications of a section of 2 μm × 2 μm. (**B**) Relative frequency distributions of receptor to receptor distances within the detected clusters of ErbB2 (**left**) and ErbB3 (**right**); for comparison see Figure 1B: (a) non-treated control; (b) after neuregulin-1 treatment of ErbB3; (c) after neuregulin-1 stimulation of ErbB3 and trastuzumab treatment of ErbB2; (d) after neuregulin-1 stimulation of ErbB3 and combined treatment of ErbB2 with trastuzumab and pertuzumab. For each curve, 21–25 cells were analyzed and merged. The number of points showed a variety of about 30–35% between the individual cells.

In Figure 2A, an example demonstrating the comparison of a wide-field image (as visible by eye through a high numerical aperture microscope objective) and a localization microscopy image (artificial pointillist image reconstructed from the position measurements of individual blinking dye molecules) is shown. The enlarged image section clearly shows the contrast and resolution improvements obtained by localization microscopy.

ErbB2 and ErbB3 receptors were partly organized in clusters of typically 70–90 nm in diameter. The distances between the clusters of the same receptor type varied between 500 and 800 nm. Within the clusters, a signal density of about 2000 signals/μm^2^ was detected. In Figure 2B, the normalized frequencies of measured distances within the clusters (after subtracting the randomly organized part of the distance distribution) are shown for the two receptor types. The data indicate an increase in cluster points and small distances of ErbB2 after stimulation of ErbB3 with neuregulin-1. This effect was not influenced by trastuzumab treatment, whereas the combination of trastuzumab and pertuzumab led to a distribution compatible with the control. In case of ErbB3, neuregulin-1 stimulation showed an increase in the frequency of smaller distances, accompanied by a cluster size reduction, as compared to the control. Trastuzumab + pertuzumab treatment showed an antagonistic effect. In contrast, trastuzumab treatment alone led to a widening of the distances and clusters.

These data indicate treatment-correlated conformational changes of receptors in the membranes of tumor cells. The results of neuregulin-1 stimulation seem to be compatible to a trafficking of ErbB3 to ErbB2 clusters for dimerization. After the application of trastuzumab alone, this seems to be suppressed, whereas the combination of trastuzumab + pertuzumab seems to suppress the dimerization (ErbB2 on the level of non-treated specimens) and the ErbB3 trafficking. Thus, localization microscopy can contribute to understanding receptor movement during the activation or inhibition of receptor dimer formation. This may be useful in cases of individually different treatment responses, in order to adapt the dose of chemo-therapeutic drugs to patients’ responses. Although such claims may so far not be verified in clinical studies, the data presented here are a first step, and demonstrate the power of such measurements on the nano-scale.

### 2.3. Chromatin Response after Smad Specific Uboquitin Protein Ligase 2 (Smurf2) Over-Expression and Cell Treatment with the Chemotherapeutic Drug Etoposide

Smurf2 (Smad specific E3 ubiquitin protein ligase 2) is an evolutionarily-conserved E3 ubiquitin ligase that belongs to the NEDD4 (neural precursor cell expressed, developmentally down- regulated gene 4) E3 family [55]. The genetic inactivation of Smurf2 led to perturbations in the chromatin structure landscape and, thereby, led to alterations in gene expression and DNA damage response. As a result, the genomic integrity in Smurf2-deficient cells was compromised, and the carcinogenic process was set in motion, leading to cell transformation and the development of a wide spectrum of tumors in the affected animals [56]. This phenotype of Smurf2-deficient mice very closely resembles tumor settings in humans, both from the point of the tumor onset and origin; the vast majority of human cancers develop at an advanced age and are of epithelial origin. Smurf2 tumor suppressor activities were associated with the chromatin-modifying enzyme RNF20. RNF20 is also an E3 ligase, responsible for histone H2B mono-ubiquitination, and thereby, responsible for the regulation of chromatin compaction, gene expression and DNA damage responses and repair [56,57,58,59]. 

Despite findings which point to Smurf2 as a potent tumor suppressor [60,61], which prevents cellular transformation and carcinogenesis, the roles of Smurf2 in human cancers appear to be more complex. A few studies conducted on immortalized and established cancer cell lines suggested that in some types of tumors Smurf2 might operate as an oncogene rather than a tumor suppressor [55,62]. It is plausible that in some types of human cancer, Smurf2 might switch roles, and promote tumor progression and chemo-resistance. The targeting of Smurf2, and molecular pathways operating under its jurisdiction, including those which regulate gene expression, DNA damage responses, and the genomic integrity maintenance, may thus have a dramatic impact on the ability of tumor cells to grow, metastasize and/or resist anticancer therapies.

**Figure 3:**
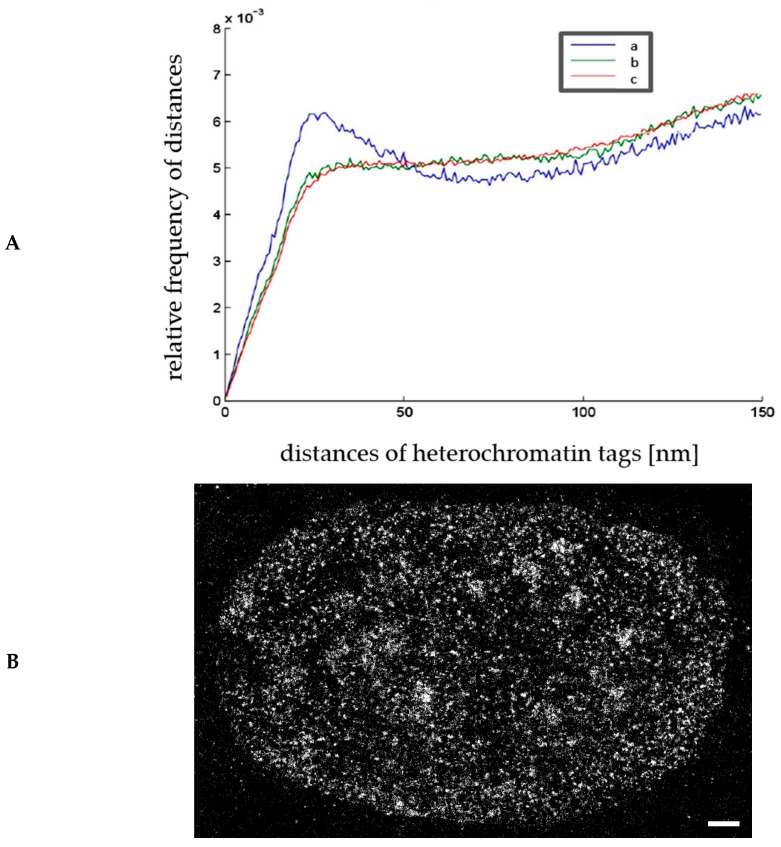
(**A**) Relative frequencies of the pair-wise distances (in nm) of heterochromatin tags (H3K9me3) labelled with a secondary antibody, carrying rhodamine red X. The curves obtained from 20 cells each (time-stacks of 3500 frames per cell) indicate compaction regions (≤100 nm) in a more randomly organized environment for wild-type U2OS cells (a). Treatment with etoposide resulted in a relaxed configuration for wild-type U2OS cells (b) and Smad specific ubiquitin protein ligase 2 (Smurf2) over-expressing U2OS cells (c). For comparison and explanation of the curves in Figure 3A, see Figure 1C; (**B**) example of an image of a cell nucleus from a U2OS cell, reconstructed from the loci matrix of heterochromatin tags (Scale bar: 1 µm).

Recently, it has been shown that Smurf2 is an essential regulator of DNA topoisomerase Iiα, which maintains genomic integrity and unaltered chromosome inheritance [63]. Smurf2 modifies the ubiquitination status of topoisomerase IIα in order to protect it from proteasomal degradation. Here, we applied etoposide (40 µM; 1 h), a topoisomerase II inhibitor that is used in the anti-cancer chemo-therapy of many types of cancer. This drug was applied to human osteosarcoma U2OS cells and the distance distributions of heterochromatin markers (i.e., H3K9me3) were studied under different conditions, in fixed specimens. In Figure 3A, the relative frequency of pair-wise distances of these heterochromatin tags are shown. In the case of wild-type cells with normal Smurf2 expression, heterochromatin shows a non-random compaction below distances of 100 nm which is well compatible to standard heterochromatin compaction, as detected by localization microscopy (e.g., [28,30,37]). An example for visualization is presented in Figure 3B, indicating in-homogenous distribution with highly condensed areas. After etoposide treatment, the cells showed a strong heterochromatin rearrangement towards de-condensation and random distribution of labelling tags.

Etoposide was also applied to U2OS cells, which were transfected with the vector containing a *Smurf2-GFP* cassette and gene for antibiotic resistance. These cells randomly integrated the DNA (*Smurf2-GFP*) into their genome, so that in addition to the endogenous *Smurf2* gene, extra copies were available. These Smurf2-GFP overexpressing cells express high levels of Smurf2-GFP fused protein, which is normally able to ubiquitinate its target proteins. Compared to the wild-type cells, etoposide treatment of Smurf2-GFP overexpressing cells showed the same effect on heterochromatin as measured by H3K9 staining. This finding suggests that overexpression of Smurf2 does not alter the distribution of heterochromatin in cells in which DNA was damaged through the etoposide/topoisomerase II/DNA double strand break axis.

Again, our data demonstrate the potential applications of localization microscopy measurements for studies of individual treatment responses.

In addition to the distribution of heterochromatin in cell nuclei, the molecular arrangements of Smurf2 were analyzed in cell modifications, in which Smurf2 was carrying a GFP as a fluorescent tag. In addition to the modification described above leading to an over-expression of Smurf2, another type of U2OS cells was used. These cells were transfected with a vector containing a *Smurf2 CG-GFP* cassette and gene for antibiotic resistance. “CG” stands for amino acid substitution (Cysteine 716 to Glycine 716) so that this mutant of Smurf2 protein is unable to ubiquitinate its substrates. The cells, which randomly integrated DNA (*Smurf2 CG-GFP*) in their genome, have in addition to endogenous *Smurf2* gene, extra copies of *Smurf2 CG-GFP*. These Smurf2 CG-GFP overexpressing cells express high levels of the Smurf2 CG-GFP protein, which is not able to ubiquitinate its target proteins. 

As a control, we used U2OS cells transfected with a vector containing a functional GFP cassette and gene for antibiotic resistance. The cells which randomly integrated DNA (GFP) into their genome express GFP tags and show normal levels of functional Smurf2 proteins.

**Figure 4:**
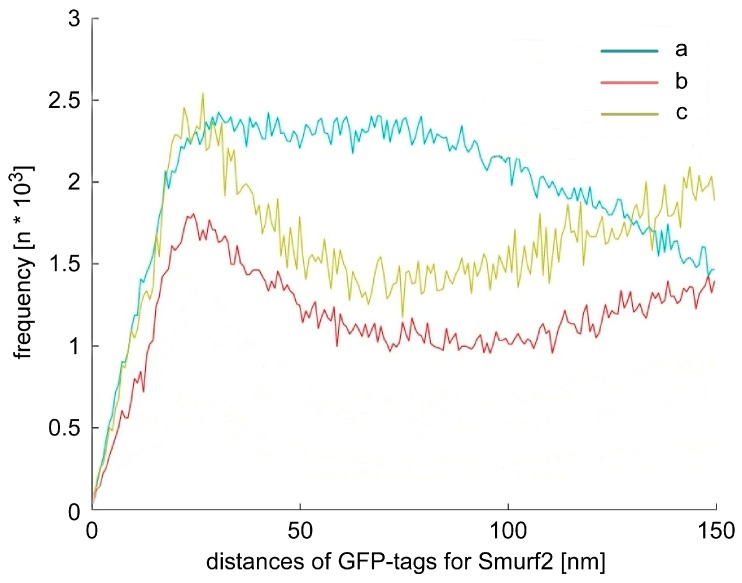
Frequency of pair-wise distances (in nm) of GFP tags for three U2OS cell line modifications: (a) cell line with GFP over-expression; (b) cell line with Smurf2-GFP over-expression; (c) cell line with Smurf2 CG-GFP over-expression. For each curve, 20 cells were measured with 3500 frames per cell. For comparison and interpretation of the curves see Figure 1C.

The results of localization microscopy measurements (Figure 4) revealed a significant difference in the organization of GFP tags, and consequently, Smurf2. The over-expressed functional Smurf2, formed conformation clusters, as is typical for activated proteins (see for instance [52]) in a more equally distributed environment of single proteins. The over-expressed non-functional Smurf2-CG showed a much stronger formation of clusters in a random environment, which may be due to vesicle formation. In contrast to the Smurf2 variants, the over-expressed GFP protein showed large aggregates. These data indicate that localization microscopy is able to discriminate differently arranged Smurf2 variants and could thus be applied to study the transition of tumor-suppressing Smurf2 in cell nuclei, to tumor-promoting Smurf2 in cellular cytosol.

### 2.4. Chromatin Response to Radiation Treatment Detected by γH2AX Foci Formation and Heterochromatin (HC) Reorganisation

**Figure 5:**
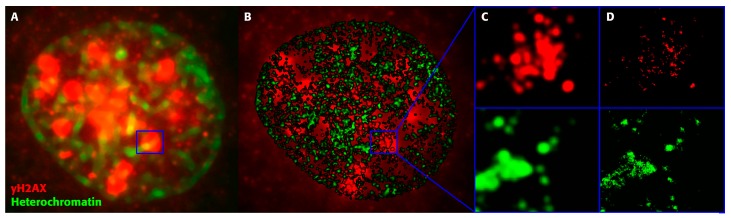
Image section of a cell nucleus of a breast cancer cell line (SKBr3) after staining of heterochromatin (green) and γH2AX foci (red) by fluorescent specific antibodies. The cells were irradiated by 1 Gy photon-radiation and the images were acquired 60 minutes after irradiation during activated repair. (**A**) wide-field fluorescence image, scale bar: 2 µm ; (**B**) localization microscopy image of the same image section, scale bar: 2 µm; (**C**) enlarged inserts of A separated in the color channels red and green, image size 2 µm × 2 µm; (**D**) enlarged inserts of B separated in the color channels red and green, image size 2 µm × 2 µm.

Since the application of ionizing radiation (also in combination with chemo-therapy) is one of the most commonly used therapeutic modalities in cancer treatment [64,65], an understanding of the mechanisms of DNA damage induction and repair in both tumor and normal cells is of fundamental interest [66,67]. On one hand, the tumor cells should be deadly injured. On the other hand, non-tumor cells have to be exposed to radiation as little as possible, so that the natural repair mechanisms can maintain cellular survival, without any secondary mutations or any other detrimental consequences [68,69,70]. Hence, repair studies have become necessary to improve tumor treatment. An example for the potential of localization microscopy in this field of research is shown in Figure 5, where the chromatin organization and the formation of γH2AX repair foci are visualized from localization microscopy data.

Such studies can be used to investigate the dynamics of H2AX phosphorylation, known as an early event after DNA strand-breaks. Further investigations by localization microscopy on analyses of γH2AX formation have indicated that the dynamics of molecule clustering during repair depends on the cell type, whereas the fundamental structural organization of these clusters seems to be similar in all cell types [71]. Moreover, using specific antibodies as tags for different repair proteins will offer the potential to study the formation of repair clusters and their internal structures at a single molecule level.

So far, we have not distinguished between γH2AX clusters attached to heterochromatin versus euchromatin. Novel studies on cluster and foci have indicated a categorization based on persistence topological analyses. γ-H2AX clusters showed similar topologies if they were adjacent to heterochromatin (HC) regions [72]. This might again offer new perspectives for analyzing patients on an individual level.

**Figure 6:**
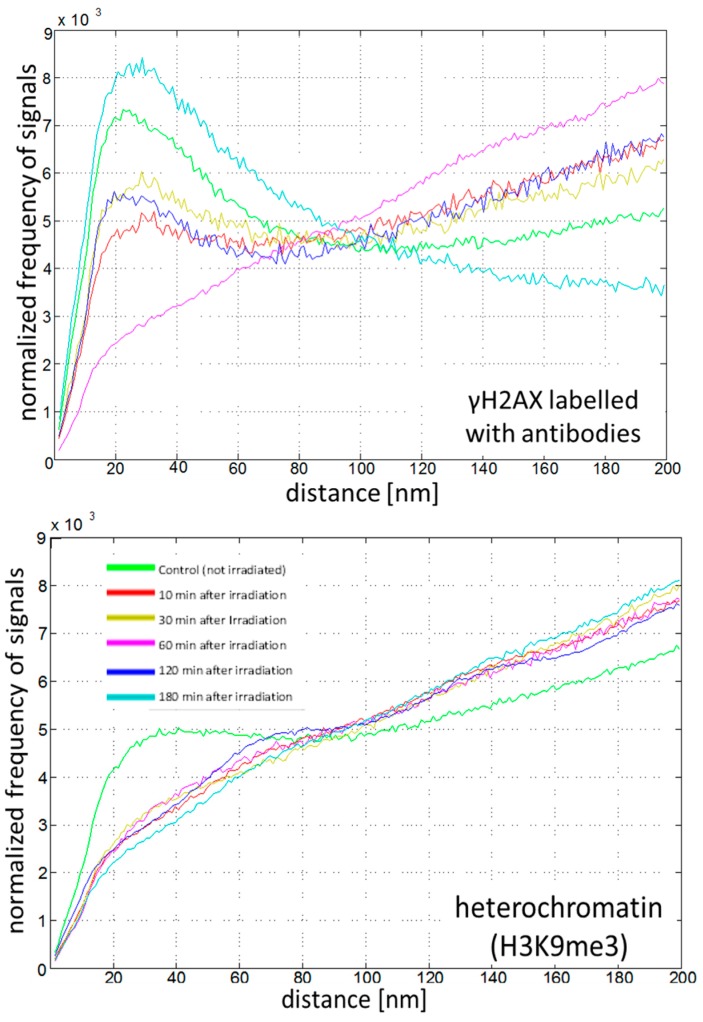
Normalized frequencies of the signal distances of fluorescence-labelled antibodies to γH2AX phosphorylation sites (upper graphs) and heterochromatin H3K9me3 methylation sites (lower graphs), as determined by localization microscopy for different repair times after exposure to 3.9 Gy ionizing radiation. The differently colored curves were obtained from 20–25 cells from aliquots that were fixed at certain time points after irradiation (see insert in the lower figure). For comparison and interpretation of the curve shapes see Figure 1; for instance, for the pink curves see Figure 1A; for the green curve in the lower figure see Figure 1C; for the light blue curve in the upper image see Figure 1B.

The repair behavior of MCF-7 breast cancer cells was analyzed at several time-points of fixation, following radiation treatment with a dose of 3.9 Gy X-rays. As an example of localization microscopy measurements, the normalized distance frequencies of γH2AX molecules and frequencies of pair-wise distances in heterochromatin tags are shown (Figure 6). From these graphs, it can be concluded that γH2AX is forming clusters within a more randomly-organized distribution of signals. At 60 min following cell irradiation, a strong increase in foci formation has occurred, and the clustering of the repair foci reaches its maximum, so that distances within a cluster, and between clusters, fall into the same range. After 180 min, the protein clusters have relaxed. Only a few tags are clustered within a very low point distribution.

The heterochromatin tags, H3K9me3, show both compact and less compact regions without treatment (see for comparison [73]). Shortly after the irradiation (10 min) the heterochromatin appears to be relaxed (see also for comparison [30]) and remained in the same state during the whole investigation period. The data indicate repair time-dependent de-compaction and re-compaction of heterochromatin and show the spatial interaction between foci formation and local chromatin re-arrangements.

### 2.5. Chromatin Response to Radiation Treatment Detected by COMBO-FISH of the ALU Consensus Regions

Radiation treatment of tumors is unfortunately mostly also associated with radiation exposure to healthy tissue cells. Sophisticated treatment planning allows the reduction of such inadvertent irradiation to a minimum so that damage may correctly be repaired. However, radiation damage effects and repair activity are also dependent on cell or tissue type as well as on the individual predispositions of patients. This is the reason for the requirement to understand the repair processes and radiation responses of different cell types and tissues well. Nevertheless, for control, it might be also relevant to measure in situ applied radiation doses, at the single cell level, by biological dosimetry.

ALU elements are short stretches of DNA (SINE = short interspersed nuclear element) which appear in repetition rates and sequence families more than 1 million times in the human genome covering about 11% of the whole DNA sequence of a cell nucleus. Thirty-seven ALU sequence families are distinguished [74], showing a close similarity. This is dependent on the probability of a consensus-sequence in the different families [75]. ALU elements are known to be involved in genome organization, gene regulation, genomic diversity [76], disease [74] and DNA double strand break repair [77,78]. ALU elements are found in several tumor suppressor genes (e.g., *BRCA1*) [79]. ALU elements are transposable elements. During the evolution of higher mammalians, they appear to have played a prominent role in genome adaptation to stress and environmental impacts [80], so they have been involved in many different functions [81,82].

Recently, we showed that ALU consensus sequences can be specifically labeled by a unique oligo-nucleotide sequence, applying a non-harsh COMBO-FISH protocol, at low temperatures [37]. After exposure to ionizing radiation, the number of labeling sites being detected by localization microscopy drops down, according to a linear–quadratic dose effect curve

*N* = *a*′*D*^2^ + *b*′*D* + *c*′ (1)

where *N* is the number of detected labelling points, *D* is the applied dose and *a*′ = 3054, *b*′ = −1.148 × 10^4^, *c*′ = 2.139 × 10^4^. At higher doses, which induce double strand breaks, this negative dose effect curve may be explained by repair effects due to single strand annealing or microhomology-mediated end joining between two ALU elements so that the labelling site is cut out. In addition, non-homologous end joining in the vicinity of ALU elements may use ALU/ALU homology, also reducing the number of target sites for our oligonucleotide-probe [77].

In the case of low doses (<500 mGy), the above-mentioned arguments may only be of minor importance. Thus, the question is whether this is also true for applied biological dosimetry via counting of ALU labelling sites. Assuming that the model conditions leading to a linear–quadratic dose-efficiency curve could be also applied to this ALU counting, the *D*^2^-term must be insignificant and negligible, so that the curve can be reduced to

*N* (low dose) = *aD* + *b* (2)

where *a* and *b* in (2) are fit parameters equal to *b*′ and *c*′ within a given error range in (1).

In Table 1 we have summarized the results of several independent experiments done with ALU-biological dosimetry, in the dose range of 0 to 1 *G*y. SkBr3 cells were exposed to 6 MeV photon-irradiation at different doses and were fixed 30 min after radiation exposure for labeling and further microscopic analyses. In case of the low dose experiments, the preparation procedure as well the localization microscopy experiments were done by different experimenters, in order to avoid personal bias.

**Figure 7:**
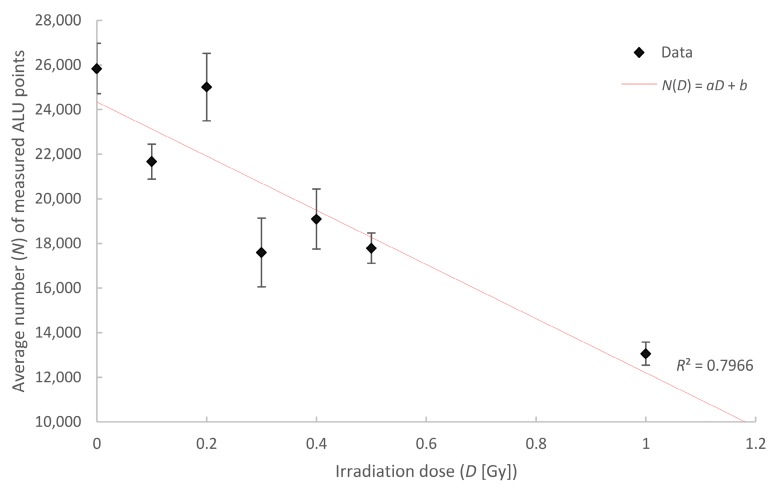
Average number of measured ALU elements relative to the radiation exposure dose. The data were detected after specimen fixation 30 min after irradiation with 6 MeV photons. The measurement data is fitted by a linear curve (*N*(*D*) = *aD* + *b*) with the parameters: *a* = −12,143, and *b* = 24,345. The error bars reflect the variations between the individual cells.

Figure 7 demonstrates the successful result of the experiments summarized in Table 1. A linear fit can be drawn through the mean values of the ALU target sites counts obtained by localization microscopy. With the fit parameter, *a* = −12,143 and *b* = 24,345, these values only differ less than ~5% from the fit values of the linear–quadratic fit in [37].

Although this quantification method shows high reproducibility and seems to be applicable over a broad range of doses, the mechanisms behind these dose effects have to be further investigated. So far, we only understand that there must be some competition of the ALU binding sites for the COMBO-FISH probes used. Recently it has been shown that ALU-RNAs are activated for RNAs of polymerase I and II [83] which may be necessary for DNA repair, also in cases of single strand damage. This may give a hint that the detected effects of ALU COMBO-FISH probes have to be studied in a complex frame of DNA repair feedback loops [84]. 

**Figure 8:**
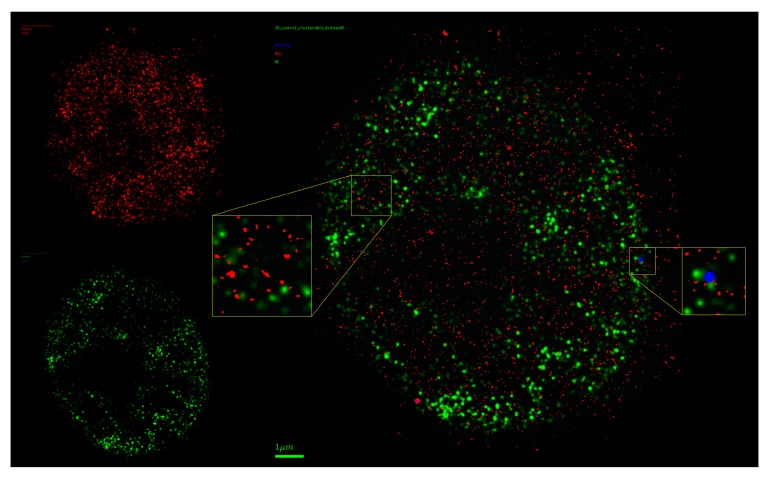
Visualization of the point matrix after two-color localization microscopy data acquisition. ALU COMBO-FISH oligonucleotides are labeled in red; heterochromatin methylation sites simultaneously labeled by antibodies (H3K9me3) are shown in green. The right image shows the merged visualization. The enlarged inserts demonstrate the exclusive arrangement of ALU sequences and heterochromatin target sites. In the whole image, only one (blue point) locus suggests a co-localization, which may be due to optical overlap (scale bar 1 µm for the merged image).

Localization microscopy of ALU labeling sites, in combination with different other DNA labels, like immunostaining of heterochromatin (Figure 8), euchromatin or GFP-labeled proteins, may deliver a tool box for such investigations of tumor genesis and treatment responses.

## 3. Materials and Methods

### 3.1. Cell Culture and Treatment

MCF-7: The cells were treated as described in detail elsewhere [52]. Michigan Cancer Foundation cell line 7 (MCF-7) cells are cultivated in Medium RPMI 1640 with 2 mM l-Glutamine, 100 U/mL Penicillin, 100 mg/mL Streptomycin, 10% fetal calf serum and 25 mM 2-(4-(2-hydroxyethyl)-1-piperazinyl)-ethan sulfonic acid (HEPES). The cells are cultivated on 19 mm × 19 mm lime glass cover slides at 37 °C and 5% CO_2_.

Stimulation of MCF-7 cells with neuregulin-1, trastuzumab and pertuzumab: At 50% confluence prior to neuregulin-1 application, the fetal calf serum (FCS) content was reduced to 0.5% followed by replacement of the complete RPMI 1640 medium without FCS for an additional 3 hours and RPMI 1640 medium without FCS but with neuregulin-1 for 15 min; and either neuregulin-1 + trastuzumab (for 15 min each), or neuregulin-1 (15 min) + trastuzumab (15 min) + pertuzumab (60 min), respectively.

SkBr3: SkBr3 cells were cultivated in McCoy’s 5A (Modified) Medium, GlutaMAX supplement with 100 U/mL Penicillin, 100 mg/mL streptomycin (all Thermofisher, Waltham, MA, USA), 10% fetal calf serum (FCS, Biochrom, Berlin, Germany) and 25 mM 2-(4-(2-Hydroxyethyl)-1-piperazinyl)-ethan sulfonic acid (HEPES) (Carl Roth, Karlsruhe, Germany). Approximately 20,000 cells were seeded on 22 mm × 22 mm lime glass cover slides (thickness class 1) (Wenzel Gläser, Berlin, Germany), in Cellstar six-well plates (Greiner Bio-One International, Frickenhausen, Germany) and grown for 48 h to reach the desired confluency before irradiation and microscopy specimen preparation. Environmental conditions were an H_2_O-saturated atmosphere of 37 °C and 5% CO_2_. For ALU dosimetry experiments (see labelling below) the cells were irradiated with 6 MeV photons (dose rate 3 Gy/min) in a medical linear particle accelerator at doses of 0.1, 0.2, 0.3, 0.4, 0.5and 1.0 Gy.

U2OS: Human osteosarcoma cells were derived from the ATCC (the American Type Culture Collection, Manassas, VA, USA). These cells were grown in a high glucose DMEM (Dulbecco’s Modified Eagle’s medium) (4,5 g/L d-Glucose, Gibco, Gaithersburg, MD, USA), supplemented with 10% (*v*/*v*) fetal bovine serum, 2 mM l-glutamine and 1% (*v*/*v*) Pen-Strep, at 37 °C with 5% CO_2_.

### 3.2. Immunostaining and COMBOinatorial Oligonucleotide Fluorescence In Situ Hybridization (COMBO-FISH)

The procedure of low temperature “COMbinatorial Oligo FISH“ (COMBO-FISH) in combination with immunostaining has been recently developed by the group of the first author is described in full detail in another issue of this journal [37]. In brief: SkBr3 cells were grown on coverslips and irradiated with different doses at 80% confluency. After washing in 1× PBS + Mg/Ca for 5 min, the cells were fixed in 4% formaldehyde (in 1× PBS + Mg/Ca) for 10 min at 37 °C followed by three washing steps in 1× PBS + Mg/Ca for 5 min. Permeablization and additional washing three times in 1× PBS + Mg/Ca on a shaker for 5 min followed. After incubation into the blocking solution for 30 min the primary antibody (anti γ-H2AX and/or anti H3K9me3) was added in a humidified environment at 37 °C for 30 min. After washing three times in 1× PBS + Mg/Ca on a shaker for 5 min the incubation with the secondary antibody followed at 37 °C for 30 min; again three times washing in 1× PBS + Mg/Ca on a shaker for 5 min and fixation in 2% formaldehyde solution (in 1× PBS + Mg/Ca) at 37 °C for 10 min. After an additional washing step, the cells were incubated in 0.1 M HCl for 10 min, washed three times in 0.05% Triton-X in 1× PBS + Mg/Ca on a shaker for 5 min, and were incubated in 2× SSC (5 min) and in 50% formamide in 2× SSC (30 min). 20 µL of COMBO-FISH probe solution was added (ALU sequence: Alexa Fluor^®^ 568-TAATCCCAGCACTTTGG). The coverslips were sealed with rubber cement (Fixogum) and put in a humidified chamber at 37 °C for 24 h. After washing three times in 2× SSC at 37 °C for 5 min, incubation in 1× PBS + Mg/Ca for 5 min, counterstaining with DAPI for 5 min, the specimen was washed twice in 1× PBS + Mg/Ca and embedded in 15 µL ProlongGold (ThermoFisher Scientific, Darmstadt, Germany). The sealed specimen can be stored at 4 °C. 

### 3.3. Localization Microscopy

For localization microscopy two different setups were applied. For the experiments in Section 2.2, Section 2.3 and Section 2.4, the setup described in [27] was used: Based on an epifluorescent microscope setup, one can switch between different solid state lasers of wavelengths 488 and 568 nm. Their power was set to 200 mW which corresponds to a power density of 10 kW/cm^2^ during localization data acquisition. The intensity of the laser(s) can be regulated via a neutral density filter wheel (i.e., a neutral grey filter) in up to 12 steps. Movable mirrors can switch it between the two modes. The emitted photons are detected by a high quantum efficiency charged coupled device (CCD) camera (Sensicam QE, PCO, Kelheim, Germany) after passing through a dichroic filter-wheel and a blocking filter-wheel (also called emission filter-wheel). The camera has a very sensitive and fast CCD-chip consisting of an array of 1376 × 1040 pixels with an area of 6.45 μm × 6.45 μm for each pixel. Together with the objective 100×/NA 1.4, this results in a pixel size of 64.5 μm × 64.5 nm for the raw data.

For the experiments described in 2.5., another setup was used [37]: Four lasers with wavelengths of 405, 491, 561, and 642 nm are available. Instead of conventional optical filters, an acousto-optic tunable filter (AOTF) is used to select the laser wavelength (405, 491, 561, or 642 nm) and set the intensity. With the AOTF, mechanical movements are circumvented so that the beam path remains stable when changing the wavelength or the intensity. In localization mode, the laser intensity was 3 kW/cm^2^ (491 nm) and 5 kW/cm^2^ (561 nm). The beam is directed into the 100× oil immersion objective lens with a numerical aperture of NA = 1.46. The image signals are acquired by an EMCCD-camera (Andor iXon Ultra 897, Belfast, UK) with 512 × 512 pixels and a pixel size of 16 µm (corresponds to 88 nm ± 2 nm object size). The exposure time was 100 ms per frame. Usually 2000 frames were acquired per cell or cell nucleus if not stated differently.

After image acquisition the loci of blinking events were determined using in-house developed computer program as recently described in [32,37,52]. The microscopy data result in a matrix that can be further processed as described in Section 2.1.

## 4. Conclusions

Lasers have found widespread applications in life sciences, especially in microscopy and have significantly contributed to modern super-resolution fluorescence techniques. In this article, we have shown some applications of fluorescence localization microscopy in combination with special procedures of nano-probing and molecular tagging, to understand molecular interactions and regulatory mechanisms in tumor initiation, progression, and treatment responses. Our intention was to address application fields on membranes in the cellular cytosol, and in the cell nucleus for advanced cancer diagnosis and novel treatment procedures for personalized medicine. The article has introduced investigations of spatial receptor arrangements in membranes and genome organization in cell nuclei on the nano-scale that may contribute to elucidating complex molecular feedback regulatory mechanisms in cells and tissues related to cancer and cancer responses. By means of the technological approaches shown here, the correlation between cell function and spatial organization of molecules or molecular complexes, can be studied, using a sophisticated tool-box of fluorescent labels, like antibodies, GFP tags, or specific oligonucleotides, and labeling strategies. Thus, nano-scaled distance measurements between bio-molecules, tagged by appropriate nano-probes can measure structures and conformations of molecular complexes and contribute to a better understanding of cellular characteristics in tumor genesis and treatment responses. Although the potential fields of application are obvious, there are still a lot of further investigations necessary for future clinical studies. 

## Figures and Tables

**Table 1:** Results of independent low dose radiation experiments using specific COMBinatorial oligo-nucleotide fluorescence in situ hybridization (COMBO FISH) labeling with a unique oligonucleotide-probe for the ALU consensus sequence.

Irradiation Dose (Gy)	Number of Cells Analyzed	Mean Number of Points	Error of the Mean (±)
control	80	25,839	1129
0.1	83	21,669	783
0.2	40	25,011	1513
0.3	40	17,594	1538
0.4	40	19,091	1347
0.5	80	17,789	676
1	38	13,061	518
